# Population density of the western burrowing owl (*Athene cunicularia hypugaea*) in Mexican prairie dog (*Cynomys mexicanus*) colonies in northeastern Mexico

**DOI:** 10.1186/s12898-016-0091-y

**Published:** 2016-08-26

**Authors:** Gabriel Ruiz Ayma, Alina Olalla Kerstupp, Alberto Macías Duarte, Antonio Guzmán Velasco, José I. González Rojas

**Affiliations:** 1 Facultad de Ciencias Biologicas, Universidad Autonoma de Nuevo Leon, Ave. Universidad s/n. Cd. Universitaria, 66455 San Nicolas de los Garza, Nuevo Leon Mexico; 2 Ley Federal del Trabajo S/N, Universidad Estatal de Sonora, Col. Apolo, 83100 Hermosillo, Sonora Mexico

**Keywords:** Chihuahuan Desert, Distance sampling, Grassland, Mexican prairie dog, Mexico, Population, Western burrowing owl

## Abstract

**Background:**

The western burrowing owl (*Athene cunicularia hypugaea*) occurs throughout western North America in various habitats such as desert, short-grass prairie and shrub-steppe, among others, where the main threat for this species is habitat loss. Range-wide declines have prompted a need for reliable estimates of its populations in Mexico, where the size of resident and migratory populations remain unknown.

**Results:**

Our objective was to estimate the abundance and density of breeding western burrowing owl populations in Mexican prairie dog (*Cynomys mexicanus*) colonies in two sites located within the Chihuahuan Desert ecoregion in the states of Nuevo Leon and San Luis Potosi, Mexico. Line transect surveys were conducted from February to April of 2010 and 2011. Fifty 60 ha transects were analyzed using distance sampling to estimate owl and Mexican prairie dog populations. We estimated a population of 2026 owls (95 % CI 1756–2336) in 2010 and 2015 owls (95 % CI 1573–2317) in 2011 across 50 Mexican prairie dog colonies (20,529 ha).

**Conclusions:**

The results represent the first systematic attempt to provide reliable evidence related to the size of the adult owl populations, within the largest and best preserved Mexican prairie dog colonies in Mexico.

## Background

**Fig. 1:**
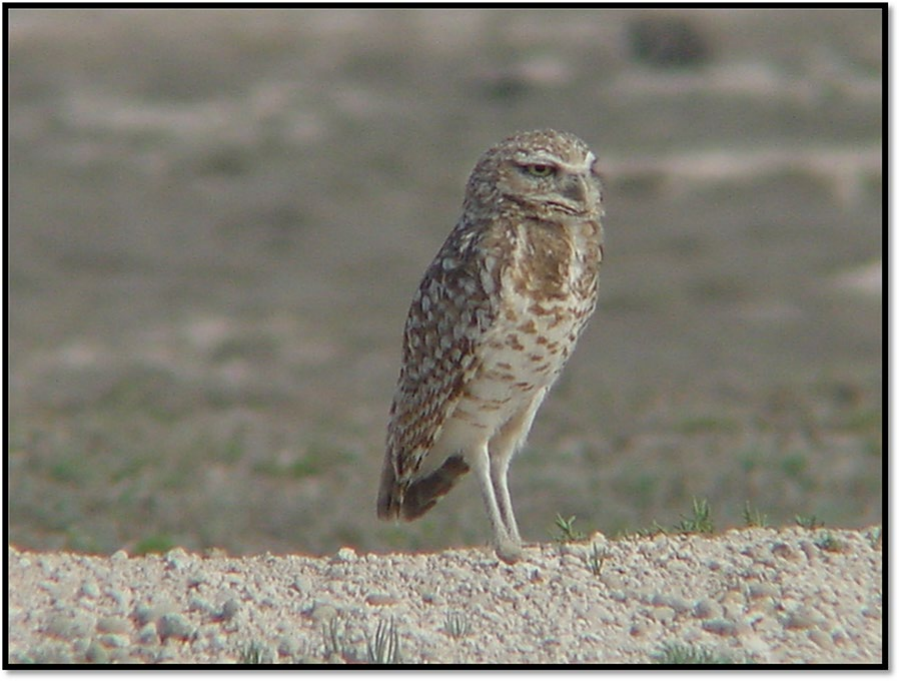
Western burrowing owl in the colony of Mexican prairie dog, in Chihuahuan Desert

Rigorous estimates of regional population size are critical for the development and assessment of avian conservation strategies, particularly for species undergoing shifts in their distribution and range. The western burrowing owl (*Athene cunicularia hypugaea*) (Fig. 1), a species with special conservation status throughout much of its range, has experienced range-wide shifts from southern Canada to northern Mexico [1]. Western burrowing owls belong to a grassland bird guild that is threatened by habitat loss [2]. The species uses open habitats such as grasslands, deserts and areas of disturbance [3]. These owls also prefer areas with discontinuous vegetation and low growth shrubs, allowing them to increase visibility for hunting, vigilance against predators and caring of burrows [4, 5].

Published data from owl populations vary within the range of distribution in North America. For example, in the 1990's population estimates of this species in Canada and the United States of America (USA) ranged from as low as 2000–20,000 to as high as 20,000–200,000 individuals [6]. In Canada, the populations have declined abruptly and even disappeared from British Columbia and Manitoba [7]. Previous reports indicate a wide variation of population trends ranging from stable in some areas in the USA and Canada, to reduced, extirpated or increasing in others [2, 7–16].

Local density estimates a range of 13–31 owls/km^2^ in Canada (Manitoba, Alberta, Saskatchewan and British Columbia) and the USA (Arizona, California, Colorado, Idaho, Iowa, Kansas, Minnesota, Montana, Nebraska, New Mexico, North and South Dakota, Oklahoma, Oregon, Texas, Utah and Washington) during the western burrowing owl breeding season indicating variation in the density estimates [1, 7, 9, 14, 17–26]. In Mexico, the federal government classifies the western burrowing owl under the category of special protection [27]. Habits of the western burrowing owl such as summer diet, prey selection, movement of juveniles, selection of nesting sites and threats remain poorly known. Densities estimated during the breeding season in 2002 in Mexico include 14.1 owls/km^2^ near Mexicali [28], 3.2 pairs/km^2^ in Yaqui-Mayo Valley, Sonora, 4.5 pairs/km^2^ in Valle del Fuerte, Sinaloa, and 4.7 pairs/km^2^ in Valle de Culiacan, Sinaloa [29]. Winter season density estimates in central Mexico include 11 owls/km^2^ in Guanajuato [30] and 5.2 owls/km^2^ in Nuevo Leon [31].

**Fig. 2:**
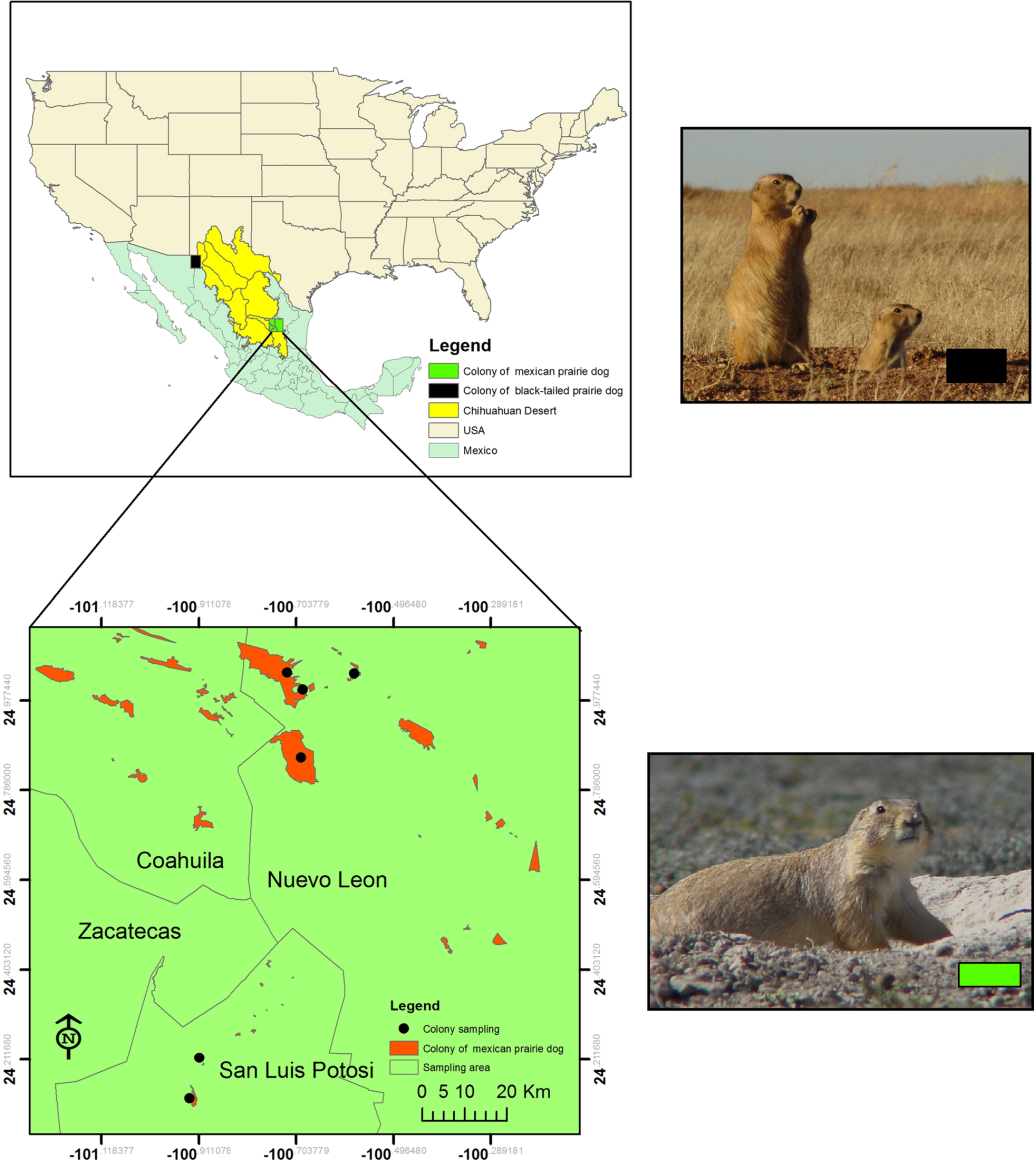
Mexican prairie dogs sampling sites, located in the Chihuahuan Desert within the states Coahuila, NL and SLP, Mexico

The western burrowing owl has been strongly associated with two species of prairie dogs in Mexico, the Mexican prairie dog (*Cynomys mexicanus*) and black-tailed prairie dog (*C. ludovicianus*) (Fig. 2) [9, 32–35]. Both of these species are federally listed in Mexico as endangered and threatened, respectively [27]. The black-tailed prairie dog is distributed from Saskatchewan in Canada to southern Montana and Nebraska in the United States to northern Chihuahua and Sonora in Mexico where the colonies are fragmented and isolated. The habitat occupied by the species of prairie dog is herbs, grasses and shrubs. Currently, the regions supporting black-tailed prairie dog colonies cover 18,500 ha [36]. The Mexican prairie dog is endemic of central and northern of Mexico within the states of Coahuila, Nuevo Leon, Zacatecas and San Luis Potosi, in colonies covering approximately 25,000 ha. These two species of dogs have lost more than 80 % of their original range [37]. Mexican prairie dog colonies provide burrows and foraging opportunities for breeding burrowing owls, which apparently keep the prairie dog population stable, despite disturbance and loss of habitat in prairie dog colonies caused by expanding agricultural and cattle grazing activities [38], the use of pesticides, collisions with vehicles, diseases, predators, and urbanization [1, 2, 7, 11, 32, 39–44].

Based on the problems and the lack of knowledge mentioned above, in this study we estimate the abundance and density of western burrowing owls in colonies of Mexican prairie dog in northeastern Mexico. Density/abundances of western burrowing owls and their association with Mexican prairie dog colonies provide relevant conservation information to ensure the long-term persistence of both species. In addition, this study can be integrated across North America to establish baseline range-wide population estimate(s) to improve our understanding of the recent range-wide shifts in owl populations.

## Methods

### Study area

Our study sites were located in Nuevo Leon (NL) and San Luis Potosi (SLP) within the Chihuahuan Desert ecoregion [45] (Fig. 2) that is part of the physiographic region known as the Mexican Plateau within the Mexican states of Coahuila, Zacatecas, NL and SLP. The semi-arid climate features temperatures ranging from 6 to 25 °C with an annual average of 16 °C [46]. Average precipitation totals 427 mm [47].

Previously, studies in NL have been conducted in the areas known as Llano de la Soledad (23°53′N, 100°42′W) and Compromiso (23°53′N, 100°42′W). These areas maintain the largest Mexican prairie dog populations, including those at Martha (25°0′N, 100°40′W), Concha (25°1′N, 100°35′W), and Hediondilla (24°57′N, 100°42′W). Western burrowing owls of SLP were studied in Llano del Manantial (24°7′N, 100°55′W) and Gallo (24°12′N, 100°54′W) in the municipality of Vanegas.

The Llano de la Soledad has been provided with several conservation designations by the NL government such as State Natural Protected Area [48], and Important Site for Bird Conservation [49]. This site hosts several vulnerable, endemic and migratory species [50, 51]. The dominant vegetation in Mexican prairie dog colonies is characterized by halophytic grassland and consists largely of *Muhlenbergia villiflora*, *Muhlenbergia repens*, *Pleuraphis mutica*, *Sporobolus airoides*, *Frankenia gypsophila* and *Dalea gypsophila*. Other coexisting plant communities include microphyllus vascular plants and rosette shrubs [31, 52–56].

From 50 colonies of prairie dogs existing in NL and SLP, nine were selected for sampling. These colonies were selected based on the following characteristics: spatial continuity of the community and a lack of fragmentation, conservation status of the site, vegetation type that was homogeneous enough to contain at least one complete transect. The sampled colonies covered about 55 % of the area available for all colonies of Mexican prairie dogs in the southern Chihuahuan Desert. Sampling was conducted between February and April in both 2010 and 2011. The transect line method was used [57]. Fifty transects (each 2 km long × 0.3 km wide and ≥0.5 km apart from each other) were traveled using the remote sampling method by the observer as described below to estimate the density of adult owls [58]. The number (*n*) of transect routes for each area was: Soledad (*n* = 28) and Compromiso (15), Marta (2) and Concha (2) in NL; Manantial (2) and Gallo (1) in SLP. We walked each transect at a constant rate using a global positioning system (GPS) to ensure a straight survey line. Owls were detected visually or with binoculars. Then, the perpendicular distance from the transect line route was measured using a laser rangefinder (15–815 m, Leica Rangemaster 900, Optics Planet, Inc. Northbrook, IL, USA). To meet the assumptions of distance sampling, only adult owls were recorded on the ground outside the burrows or without movement [58, 59]. If the bird under observation moved because of the presence of the observer, registering the perpendicular distance was performed at the original site without the observer leaving their sighting transect travel line. Those adult owls flying with an unknown initial location were not documented. To reduce bias and avoid an overestimation of population density, only adult owls were recorded. Considering the extreme desert climate, personal observations made during previous years and different criteria of previous authors related to the activities of owls, the field observations were conducted from 0600 to 1200 h [20, 23, 60, 61].

### Data analysis

We used program *DISTANCE ver. 6.0* to obtain western burrowing owl density estimates from distance sampling [62]. *DISTANCE* calculates density and abundance using modeling detection probability as a function of the perpendicular distance to the transect in a series of monotonic models. Several standard detection functions (uniform, half-normal, or hazard-rate) with cosine series adjustment were evaluated using the Akaike information criterion (AIC). We used the AIC to select the model with the most parsimonious detection function in *DISTANCE* [58, 59, 63]. We pooled all data to estimate a single detection function (probability of detection, *g* (*x*), at a given distance (*x*) from the transect) because we did not anticipate effects of environmental features on detection, such as *age* (adult) and factor *STATE* (levels: NL and SLP). We considered serial adjustments of one to three parameters. We did not truncate the data because the frequencies of long distances observations were better maintained in this manner [4].

The estimator of density ($\hat{D}$) is given by the expression:

$$\hat{D}\, = \,\frac{{\hat{n}\hat{f}(0)}}{2L}$$

where $\hat{f}(0)$ is the probability density function of detection distances from the line evaluated at zero distance, calculated in *DISTANCE* as the average number of individuals per detection [62]. The standard error of density SE $(\hat{D})$, assuming a Poisson distribution of counts, can be approximated using the delta method as follows [58]:

$$SE(\hat{D})\, = \,\hat{D}\sqrt {\frac{1}{n} + \frac{{Var(\hat{f}(0))}}{{(\hat{f}(0))^{2} }}} ,$$

where $Var(\hat{f}(0))\, = \,(SE(\hat{f}(0))^{2}$ also is a direct output of *DISTANCE*. The component cluster size was omitted from the above formulas because virtually all detections were individual records. Estimates of density and their standard errors were used to test statistical differences in density between states and years using a Wald test [64]. Values are presented as mean ± SE.

Overall estimates of western burrowing owl density (and their SE) at the nine sampled colonies (9620 ha) were obtained by pooling detection distance data by year. These estimates were then multiplied by the total area of the 50 colonies of the Mexican prairie dog described for the southern part of the Chihuahuan Desert to provide yearly estimates of owl population size through the range of Mexican prairie dog: 38 in NL (19,802 ha) and 12 in SLP (727 ha) [37]. On average, 55 % of the surface reported for the Mexican prairie dog colony complex was sampled [37].

## Results

### Density and population size

Colonies were stable during the years 2010–2011 and were not destroyed or fragmented (agriculture, livestock) during this time. During the 2010 and 2011 sampling periods, 235 detections of at least one owl were recorded. The estimates of western burrowing owl density in the 50 prairie dog colony complex were 9.8 ± 1.0 ind/km^2^ (CV 0.107) in 2010 and 9.8 ± 1.0 ind/km^2^ (CV 0.108) in 2011. The owl density estimate for NL was 8.8 ± 1.0 ind/km^2^ (CV 0.114) in 2010 and 7.3 ± 0.9 ind/km^2^ (CV 0.123) in 2011. For SLP, the owl population density was 26.7 ± 6.2 ind/km^2^ (CV 0.236) in 2010 and 47 ± 8.4 ind/km^2^ (CV 0.180) in 2011 (Table 1). No significant differences were found among western burrowing owl densities (Wald test, *p* = 0.431) and the paired states of NL (*p* = 0.967) and SLP (*p* = 0.635).

**Table 1:** Western burrowing owl population density between 2010 and 2011 in Mexican prairie dog colonies in NL and SLP, Mexico

Model^a^	D^b^	N^c^	Estimated density (owl/ha)			CV^f^	No. colony^g^	Area (ha)
			Average	95 % IC^d^	95 % IC^e^			
Global								
2010								
	0.1	119	949	788	1039	0.107	9	9620*
			2026	1765	2326		50	20,529**
2011								
	0.09	116	944	783	1035	0.108	9	9620*
			2015	1753	2317		50	20,529**
NL								
2010								
	0.08	100	809	698	937	0.114	6	9170*
			1747	1508	2024		50	20,529**
2011								
	0.074	82	678	578	794	0.124	6	9170*
			1464	1248	1716		50	20,529**
SLP								
2010								
	0.26	19	118	37	87	0.236	3	450*
			190	60	141		12	727**
2011								
	0.47	34	211	48	167	0.180	3	450*
			341	170	397		12	727**

^*^Total area of sampled colonies

^**^Total area of colonies in SLP and NL

^a^Model base done AIC criteria: half-normal + cosine

^b^Western burrowing owl density (owl/ha)

^c^Total number of detections in both years

^d^Upper confidence intervals

^e^Lower confidence intervals

^f^Variation coefficient for the estimated density

^g^Number of Mexican prairie dog colonies

Applying the overall yearly estimates of western burrowing owl density to the entire area of the 50-colony complex of prairie dogs in NL and SLP resulted in a population size of 2026 (CV 0.173) in 2010 and 2015 (CV 0.213) in 2011. For colonies in NL, an average population size of 1747 (CV 0.178) was obtained in 2010 and 1464 (CV 0.218) for 2011, while in SLP, population estimates were between 190 (CV 0.312) and 341 (CV 0.322) for each year.

## Discussion

To date, many density estimates have been made for the western burrowing owl in Canada and the USA, with quite variable results [1, 6, 7, 9, 11–15, 20–23, 25, 26]. The resulting variation in the population sizes can be attributed to the size of sample area, methodology, analytical precision, timing, observer skill, and so on; these have contributed to an inexact picture of the density of the western burrowing owl populations [6, 15]. Therefore, a comparison of our results with those of the USA and Canada could be difficult.

During the last 30 years, the North American Breeding Bird Survey has estimated a negative trend for the western burrowing owl population for Canada and the USA. Similarly, the United States Geological Survey (2014) has reported the same negative trend in the Chihuahuan Desert region [16].

Even though Mexico has not established systematic surveys that allow the establishment of a population trend, some studies (the present one included) can form the basis to achieve this goal of documenting population trends in the future.

In NL and SLP, the average density of breeding pairs (9.8 ind/km^2^) in 2010 and 2011 is greater than that reported by Macias-Duarte in Sonora (6.4 ind/km^2^) and similar to the Sinaloa average (9.2 ind/km^2^) [29]. However, in Baja California, Itubarria-Rojas reported an average of 14.1 ind/km^2^, which is a value higher than that determined by the present study [28]. This difference could be caused by the habitat quality among sites as reported in NL and SLP where burrow competition is related to the abundance of prairie dogs per colony or Baja California where the owls use irrigation canals to create burrows.

Our overall estimates of population size for western burrowing owls reveal the relative importance of Mexican prairie dog colonies to owl population viability. No previous data related to population size estimates in owls is available for the study area. However, the precision of these estimates must be taken with caution because of the variability between sites. However, we believe the extrapolation is correct because we sampled over 55 % of the current area with the active prairie dog colonies in both states. The range of the western burrowing owl in northeastern (NL, SLP, and Coahuila) Mexico includes viable colonies of Mexican prairie dogs. These areas provide an optimal habitat for the prairie dogs to feed on grasses and this contributes to a low height of herbaceous plants and allows the owls greater visual access to the foraging area. This species uses prairie dog colonies as a place for nesting, protection against climatic factors (extreme temperatures, flooding by rain, and strong winds). The owls also respond to alarm calls by prairie dogs, alerting them to the presence of predators. The western burrowing owl colonies in Mexico have declined from 88 colonies to 53, equivalent to a loss of 37 % in 10 years (1992–2003) [37, 38].

Many of the problems in northeastern Mexico that involve the western burrowing owl are directly related to loss of habitat from agriculture, but some direct mortality has been caused by collisions with vehicles. However, another possible cause of morbidity and mortality could be the direct or incidental (by bioaccumulation) exposure to pesticides used in neighboring areas.

## Conclusions

These results represent the first systematic effort to address the conservation status of the western burrowing owl populations in Mexican prairie dog colonies located in northeastern Mexico. This geographic area is considered to contain the largest preserved Mexican prairie dog colonies in the country and deserves attention from the scientific and conservation communities. Furthermore, these results contribute new information to our understanding of the population dynamics of this kind of species across North America, and highlight the urgent need to preserve grasslands, particularly those in the southern part of the Chihuahuan Desert, which harbor many bird species cataloged as threatened or endangered.

## Abbreviations

### Regions

USA: United States of America; NL: Nuevo Leon; SLP: San Luis Potosi.

### Units

km: kilometers; ind/km^2^: individual per square kilometer; ha: hectare; *n*: number of line transects; m: meters; mm: millimeter; °C: celsius; hr: hours.

### Statistical

*gx*: probability of detection; *x*: given distance; ± SE: standard error; *CV*: coefficient variation; *p*: probability; AIC: akaike information criterion; IC: confidence intervals.

### Orientation

N: north; W: western; GPS: global positioning system.

## References

[1] Macias-Duarte A, Conway CC (2015). Distributional changes in the western burrowing owl (*Athene cunicularia hypugaea*) in North America from 1967 to 2008. J Raptor Res.

[2] ACA. Commission for Environmental Cooperation. In: North America conservation action plan (*Athene cunicularia hypugaea*). Commission for Environmental Cooperation. Printed in Canada; 2005. p. 1–55. http://www.cec.org Accessed 15 Apr 2009.

[3] Clark RJ (1997). A review of the taxonomy and distribution of burrowing owl (*Speotyto cunicularia*). J Raptor Res.

[4] Coulombe HN (1971). Behavior and population ecology of the burrowing owl, *Speotyto cunicularia*, in the Imperial Valley of California. Condor.

[5] Howell GR, Webb S. A Guide to the birds of Mexico and Central America. Oxford University Press; 1995. pp. 364.

[6] James PC, Espie RHM (1997). Current status of the burrowing owl in North America: an agency survey. J Raptor Res.

[7] COSEWIC. Assessment and update status report on burrowing owl *Athene cunicularia* in Canada. Committee on the status of endangered wildlife in Canada. Ottawa; 2006. pp. 31.

[8] Desante DF, Ruhlen ED, Adamany SL, Butron KM, Amin S. A census of burrowing owls in central California in 1991. In: Lincer J, Steenhof K, editors. The burrowing owl, its biology and management including the proceedings of the first international burrowing owl symposium; 1997.

[9] Desmond MJ, Savidge JA (1996). Factors influencing burrowing owl (*Speotyto cunicularia*) nest densities and numbers in western Nebraska. Am Midl Nat.

[10] Clayton KM, Schmutz JK (1999). Is the decline of burrowing owls *Speotyto cunicularia* in prairie Canada linked to changes in great plains ecosystems?. Bird Conserv Int.

[11] Arrowood PC, Finley CA, Thompson C (2001). Analyses of burrowing owl populations in New Mexico. J Raptor Res.

[12] Korfanta NM, Ayers LW, Anderson SH, McDonald DB (2001). A preliminary assessment of burrowing owl status in Wyoming. J Raptor Res.

[13] Sheffield SR, Howery M (2001). Current status, distribution, and conservation of the burrowing owl in Oklahoma. J Raptor Res.

[14] Murphy RK, Hasselbland DW, Grondahl CD, Sidle JG, Martin RE, Feed DW (2001). Status of the burrowing owl in North Dakota. J Raptor Res.

[15] Klute DS, Green TM, Howe WH, Jones ST, Shaffer JL, Sheffield SR, Zimmerman TS. Status assessment and conservation plan for the western burrowing owl in the United States. US Department of Interior, Fish & Wildlife Service, Biological Technical Publication FWS/BTP-R6001-2003, Washington, D.C.; 2003.

[16] Sauer JR, Hines J E, Fallon JE, Pardieck KL, Ziolkowski DJ. Link the North American breeding bird survey, results and analysis 1966–2013. Version 01.30.2015 USGS Patuxent Wildlife Research Center, Laurel. USA; 2014.

[17] Butts KO, Lewis JC (1982). The importance of prairie dog towns to burrowing owls in Oklahoma. Proc Okla Acad Sci.

[18] Enriquez-Rocha P, Rangel-Salazar DW (1993). Presence and distribution of Mexican owls: a review. J Raptor Res.

[19] Trulio L (1997). Burrowing owl demography and habitat use at two urban sites in Santa Clara County, California. J Raptor Res.

[20] Conway CJ, Simon JC (2003). Comparison of detection probability associated burrowing owl survey methods. J Wildl Manag.

[21] Desante DF, Ruhlen ED, Rosenberg DK (2004). Density and abundance of burrowing owls in the agricultural matrix in the Imperial Valley. Stud Avian Biol.

[22] Manning JA. Burrowing owl population size in the Imperial Valley, California: survey and sampling methodologies for estimation. Final report to the Imperial irrigation district, Imperial, California, USA; 2009. http://www.iid.com/Modules/ShowDocument.aspx?documentid=8172. Accessed 15 Apr 2009.

[23] Tipton HC, Doherty PF, Dreitz VJ (2009). Abundance and density of mountain plover (*Charadrius montanus*) and burrowing owl (*Athene cunicularia*) in eastern Colorado. Auk.

[24] Berardelli D, Desmond JM, Murray L (2010). Reproductive success of burrowing owls in urban and grassland habitats in southern New Mexico. Wilson J Ornithol.

[25] Crowe D, Longshore K. Population status and reproduction ecology of the western burrowing owl (*Athene cunicularia hypugaea*) in Clark Contry, Nevada. Report final 2005.USGS-582-P. United States Geological Survey; 2010. pp. 31.

[26] Wilkerson RL, Sigel RB (2011). Distribution an abundance of western burrowing owls (*Athene cunicularia hypugaea*) in southeastern California. Southwest Nat.

[27] NOM-059-SEMARNAT-2010. Protección ambiental-Especies nativas de Mexico de flora y fauna silvestres-categorias de riesgo y especificaciones para su inclusion, exclusion o cambio-lista de especies en riesgo. DIARIO OFICIAL DE LA FEDERACION; 2010. http://www.profepa.gob.mx/innovaportal/file/435/1/NOM_059_SEMARNAT_2010.pdf. Accessed 30 Dec 2010.

[28] Itubarria-Rojas H. Estimacion de abundacia y afinidad de habitat del tecolote llanero (*Athene cunicularia*) en el Valle de Mexicali California y Sonora, Mexico. Universidad Autonoma de Guadalajara. Facultad de Ciencias Quimicas y Biologicas. Tesis de licenciatura. Guadalajara, Jalisco, Mexico; 2002. pp. 36.

[29] Macias-Duarte A. Change in migratory behavior as possible explanation for burrowing owl population declines in northern latitudes. The University of Arizona. School of Natural Resources and the Environmental. PhD Thesis. USA; 2011. pp. 145.

[30] Valdez-Gomez HE, Holroyd GL (2000). The burrowing owl, habits and distribution center in western Mexico. Boletin de la Sociedad de Ciencias Naturales de Jalisco.

[31] Cruz-Nieto MA. Ecologia invernal de la lechuza llanera (*Athene cunicularia*), en los pastizales ocupados por los perritos llanero Mexicano (*Cynomys mexicanus*), Nuevo Leon, Mexico. Universidad Autonoma de Nuevo Leon. Facultad de Ciencias Biologicas. Laboratorio de Ornitologia. San Nicolas de los Garza. Tesis Doctorado en Ciencias; 2006. pp. 118.

[32] Desmond MJ, Savidge JA, Eskridge KM (2000). Correlations between burrowing owl and black-tailed prairie dog declines: a 7-year analysis. J Wild Manag.

[33] Griebel RL, Savidge JA (2003). Factors related to body condition of nestling burrowing owls in Buffalo Gap National Grassland, South Dakota. Wilson J Ornithol.

[34] McNicolle JL. Burrowing owl (*Athene cunicularia*) nest site selection in relation to prairie dog colony characteristics and surrounding land-use practices in Janos, Chihuahua, Mexico. Las Cruces, New Mexico, New Mexico State University. Ms Thesis. USA; 2005. pp. 54.

[35] Ruiz-Ayma G. Exito reproductive, entrega de presas y dieta del tecolote (*Athene cunicularia hypugaea*) en el complejo de la colonias de perrito de la pradera Mexicano (*Cynomys mexicanus*) en Galeana, Nuevo Leon, Mexico. Universidad Autonoma de Nuevo Leon. Facultad de Ciencias Biologicas. Tesis Maestria en Ciencias. Mexico; 2009. pp. 85.

[36] Ceballos GOG. Los mamiferos silvestres de Mexico. Ed. Fondo de Cultura Economica de España; 2009. pp. 986.

[37] Carrera MMA. Situacion actual, estrategias de conservacion y bases para recuperacion del perrito llanero mexicano (*Cynomys mexicanus*). Universidad Autonoma de Mexico. Tesis Maestria en Ciencias. Mexico; 2008. pp. 72.

[38] Scott-Morales L, Estrada E, Chavez-Ramirez M, Cotera M (2004). Continued decline in geographic distribution of Mexican prairie dog (*Cynomys mexicanus*). J Mammal.

[39] Green GA, Anthony RG (1989). Nesting success and habitat relationships of burrowing owl in the Columbia Basin. Oregon Condor.

[40] Haug EA, Millsap BA, Martell MS. Burrowing owl (*Speotyto cunicularia*). In: Poole A, Gill F, editors, The birds of North America, No. 61. Academy of Natural Sciences, Philadelphia, and American Ornithologists’ Union, Washington, DC; 1993. pp. 20.

[41] Sheffield SR. Current status, distribution, and conservation of the burrowing owl (*Speotyto cunicularia*) in Midwestern North America. 1997. In: Duncan JR, Johnson DH, Nicholls TH, editors. Biology and conservation of owls of the Northern Hemisphere, USDA Forest Service, General Technical Report NC-190. North Central Forest Experiment Station, St. Paul, Minnesota;1997. pp. 399–407.

[42] Wellicome TI. Effects of food on reproduction in burrowing owl (*Athene cunicularia*) during three stages of the breeding season. University of Alberta. Edmonton. PhD Thesis. Canada; 2000. pp. 113.

[43] Holroyd GR, Rodriguez RE, Sheffield S (2001). Conservation of the burrowing owl in western North America, challenges and recommendations. J Raptor Res.

[44] McDonald D, Korfanta M, Lantz SJ. The burrowing owl (*Athene cunicularia*): a technical conservation assessment Wyoming, USDA Forest Service, Rocky Mountain Region; 2004.

[45] CONABIO. Comision Nacional para el Conocimiento y Uso de la Biodiversidad. Mexico; 2008. http://www.conabio.gob.mx/informacion/metadata/gis/ecort08gw.xml?_xsl=/db/metadata/xsl/fgdc_html.xsl&_indent=no. Accessed 26 Sept 2009.

[46] CONAGUA. Comisión Nacional del Agua. Consulta base de datos. Distrito Federal, Mexico. http://www.smn.cna.gob.mx/es/emas. Accessed 15 Sept 2009.

[47] Instituto Nacional De Estadística Geografia e Informatica. Conjunto de datos vectoriales de la carta de uso del suelo y vegetacion, escala 1:250,000, Serie III. INEGI. Mexico; 2005.

[48] Periodico Oficial. Monterrey, N. L., Gobierno Constitucional del Estado Libre y Soberano de Nuevo Leon, Mexico. Tomo CXXXIX; 2002.

[49] WHSRN. Designación de sitio en categoria de importancia internacional para la conservacion de aves playeras de la red hemisferica de reservas para aves playeras. 2005. http://www.whsrn.org/site-profile/llano-de-la-soledad. Accessed 03 Dec 2014.

[50] Macias-Duarte A, Panjabi AO, Pool D, Youngberg E, Levandoski G. Wintering grassland bird density in Chihuahuan Desert grassland priority conservation areas, 2007–2011. Rocky Mountain Bird Observatory, Brighton, CO, RMBO Technical Report INEOTROP- MXPLAT-10-2; 2011. pp. 164.

[51] Del Coro-Arizmendi, Marquez VL. Areas de importancia para la conservacion de las aves, CONABIO & Fondo Mexicano para la Conservacion de la Naturaleza; 2000. http://www.conabioweb.conabio.gob.mx/aicas/doctos/NE-36.html. Accessed 03 Dec 2010.

[52] Johnston MC (1963). Past and present grassland of southern Texas and northeastern Mexico. Ecology.

[53] Rojas MP. Generalidades sobre la vegetacion del estado de Nuevo Leon y datos acerca de su flora. Facultad de Ciencias. Mexico D.F, Universidad Nacional Autonoma de Mexico; 1965. pp. 124.

[54] Rivera RE. Caracterizacion y productividad invernal de tres areas de pastizal habitat para la lechuza llanera (*Athene cunicularia*) en el Municipio de Galeana, Nuevo Leon; Mexico. Universidad Autonoma de Nuevo Leon. Facultad de Ciencias Biologicas. Tesis licenciatura. México; 2006. pp. 72.

[55] Garcia RAM. Habitat reproductivo del gorrion de Worthen (*Spizella wortheni*) en cuatro localidades del noreste de Mexico. Universidad Autonoma de Nuevo Leon. Facultad de Ciencias Biologicas. Tesis licenciatura. México; 2008. pp. 71.

[56] Martinez RLM. Caracterizacion de los sitios de anidación del gorrion de Worthen (*Spizella wortheni*) en los estados de Nuevo Leon y Coahuila de Zaragoza, Mexico. Universidad Autonoma de Nuevo Leon. Facultad de Ciencias Biologicas. Tesis licenciatura. México; 2009. pp. 47.

[57] Ralph CJ, Geupel GR, PYLE P, Martin TE, Desante DF, Milá B. Manual de metodos de campo para el monitoreo de aves terrestres. General Technical Report PSW–GTR–159, USDA Forest Service, Albany; 1996.

[58] Buckland ST, Anderson DR, Burnham KP, Laake JL, Borchers DL, Thomas L (2001). Introduction to distance sampling.

[59] Buckland ST, Anderson DR, Burnham KP, Laake JL, Borchers DL, Thomas L (2004). Introduction to distance sampling.

[60] Manning JA (2011). Factors affecting detection probability of burrowing owls in southwest agroecosystem environments. J Wild Manag.

[61] Manning JA, Kaler RSA (2011). Effects of survey methods on burrowing owl behaviors. J Wild Manag.

[62] Thomas L, Buckland ST, Rexstad EA, Laake JL, Strindberg S, Hedley SL, Bishop JRB, Marques TA, Burnham KP (2010). Distance software: design and analysis of distance sampling surveys for estimating population size. J Appl Ecol.

[63] Burnham KP, Anderson DR (2002). Model selection and multimodel inference: a practical information-theoretic approach.

[64] MCulloch CE, Searle SR, Neuhaus JM. Generalized, linear and mixed models. 2nd ed. Wiley: New York; 2008.

[65] Ruizayma G, Olallakerstupp A, Maciasduarte A, Antonio G, Gonzalezrojas JI. Data from: population density of the burrowing owl (*Athene cunicularia hypugaea*) in Mexican prairie dog (*Cynomys mexicanus*) colonies at northeastern México. BMC Ecol. http://dx.doi.org/10.5061/dryad.pm362. 10.1186/s12898-016-0091-y PMC5002120 27566259

